# Rules for the Leg Coordination of Dung Beetle Ball Rolling Behaviour

**DOI:** 10.1038/s41598-020-66248-7

**Published:** 2020-06-09

**Authors:** Binggwong Leung, Nienke Bijma, Emily Baird, Marie Dacke, Stanislav Gorb, Poramate Manoonpong

**Affiliations:** 1 Bio-inspired Robotics and Neural Engineering Lab, School of Information Science and Technology, Vidyasirimedhi Institute of Science and Technology, Rayong, 21210 Thailand; 2 Functional Morphology and Biomechanics, Zoological Institute, Kiel University, Kiel, 24118 Germany; 3 Division of Functional Morphology, Department of Zoology, Stockholm University, Stockholm, SE 10691 Sweden; 4 Department of Biology, Lund Vision Group, Lund University, Sölvegatan 35, 223 62 Lund, Sweden; 5 Embodied AI and Neurorobotics Lab, SDU Biorobotics, The Mærsk Mc-Kinney Møller Institute, The University of Southern Denmark, Odense, 5230 Denmark

**Keywords:** Animal behaviour, Biomechanics

## Abstract

Dung beetles can perform a number of versatile behaviours, including walking and dung ball rolling. While different walking and running gaits of dung beetles have been described in previous literature, little is known about their ball rolling gaits. From behavioural experiments and video recordings of the beetle *Scarabaeus (Kheper) lamarcki*, we analysed and identified four underlying rules for leg coordination during ball rolling. The rules describe the alternation of the front legs and protraction waves of the middle and hind legs. We found that while rolling a ball backwards, the front legs are decoupled or loosely coupled from the other legs, resulting in a non-standard gait, in contrast to previously described tripod and gallop walking gaits in dung beetles. This provides insight into the principles of leg coordination in dung beetle ball rolling behaviour and its underlying rules. The proposed rules can be used as a basis for further investigation into ball rolling behaviours on more complex terrain (e.g., uneven terrain and slopes). Additionally, the rules can also be used to guide the development of control mechanisms for bio-inspired ball rolling robots.

## Introduction

Insect locomotion is a complex process which needs to coordinate many motor units in real time while simultaneously interacting with the environment. To gain a better understanding of how insects move over the ground, many principles of terrestrial locomotion have been investigated^1^. Leg coordination is one of the principles which has been widely studied in cockroaches^2–6^, stick insects^7–16^, and ants^17^. Various gaits underlying leg coordination have been found, including tripod, tetrapod, and metachronal wave. Different rules or hypotheses have been proposed to describe the leg coordination principle, as a basis for more complex behaviours. While the leg coordination in locomotion has been studied extensively, little research has been done on the leg coordination of large object manipulation and transportation (i.e., a combination of locomotion and object manipulation), like the ball rolling behaviour of dung beetles.

**Figure 1:**
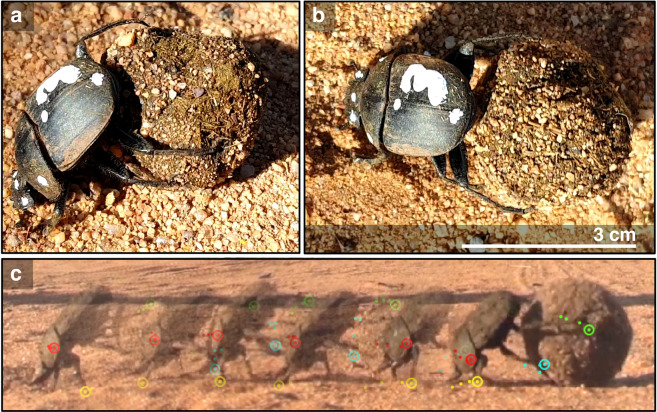
Dung beetle’s ball rolling behaviour. (**a**) Side view of ball rolling behaviour. (**b**) Top view of ball rolling behaviour. (**c**) Side view of a sequence of the ball rolling behaviour. The positions of the body and the front, middle, and hind legs of a dung beetle are marked with red, yellow, turquoise-blue, and green dots, respectively.

Dung beetles perform complex versatile behaviours including flying, walking, manipulating or creating a large dung ball, and dynamically pushing or transporting it^18^. Typically, dung beetles walk with a tripod gait but at least one species occasionally gallops^19^. To form a ball from a large dung pile, they mainly use their front legs and head while their middle and hind legs are used for stability on the dung^20^. Their unique and complex ball rolling behaviour requires simultaneous leg coordination to perform multiple actions such as walking, pushing the ball, and retaining balance. Specifically, the dung beetle walks backwards with the front legs on the ground, and the middle and hind legs are used to roll and stabilize the ball (Fig. 1). While Matthews^21^ roughly describes the front, middle, and hind leg movements of the dung beetle’s rolling behaviour, details and rules of their leg coordination for the particular ball rolling gait pattern remain unknown. A description of the leg coordination rules can provide a guideline to 1) understand how dung beetles simultaneously and dynamically interact with different substrates (e.g. ground and ball), and 2) develop bio-inspired robot control technology for achieving multiple functions (walking and transporting or rolling an object), as well as solving complex motor control problems in many degrees of freedom systems.

From this perspective, this work presents a detailed investigation into the ball rolling behaviour of *Scarabaeus (Kheper) lamarcki*. Behavioural experiments and video recordings are performed to extract the ball rolling gaits and leg trajectories, using a statistical approach to analyse the gait patterns and identify the underlying rules for describing the patterns. Four main generic rules are proposed, which can also be applied to other ball rolling conditions, e.g., rolling a dung ball with a lighter weight. We also compare the swing and stance phases of different ball rolling conditions (i.e., normal ball weight and lighter weight). Additionally, we discuss gait in relation to locomotion and object manipulation (i.e., ball pushing).

## Methods

### Ball rolling behavioural data collection

The species of the dung beetle used in this study is *Scarabaeus (Kheper) lamarcki*. The ball rolling experiments were conducted outdoors on sandy ground. The rolling behaviour was recorded using a Sony RX10III video camera at 50 frames per second for top (Fig. 1b) and side (Fig. 1c) views. Leg trajectories and ball rolling gaits were extracted from the side and top views, respectively, using video tracking programs called Tracker and ImageJ^22^ with the MTrackJ plugin. We used a well-established tracking technique^20^ to track the leg tip position of the front, middle, and hind legs relative to the dung beetle body to create the leg trajectories. To create a ball rolling gait diagram, we tracked each leg of the beetle frame by frame to see whether or not the leg was touching the ground. Thus, each leg has information on the stance and swing phases. Gait patterns were then created from the information on the stance and swing phases of each leg. In addition, the stance and swing phases were extracted from multiple ball rolling runs. In the light ball condition, we followed the same procedure to extract the ball rolling gait from the video recordings.

### Ball rolling gait pattern analysis

The purpose of gait pattern analysis is to find the underlying leg coordination pattern from the ball rolling gait by specifying the relationship between the movement of each leg. Thus, we analysed the rolling gaits using leg gait and leg swing phase similarity. The following formulae are applied to perform statistical analysis:

1 $$Leg\,gait\,similarity\, \% ({l}_{1},{l}_{2})=\frac{Total\,similar\,state({l}_{1},{l}_{2})\times 100}{Total\,time}$$

2 $$Leg\,swing\,phase\,similarity\, \% ({l}_{1},{l}_{2})=\frac{Total\,similar\,swing\,state\,({l}_{1},{l}_{2})\times 100}{Total\,time}$$

To define leg gait similarity between a pair of legs (${l}_{1}$ and ${l}_{2}$) in Eq. (1), we first count the time frame where the state of ${l}_{1}$ is the same as ${l}_{2}$. This value is then divided by a full ball rolling gait cycle (*total time*) and multiplied by one hundred to get the percentage value. The higher the percentage, the more likely the gait of the leg pair is symmetrical. On the other hand, the lower the percentage, the more likely the gait of the pair of the legs is asymmetrical. The leg swing phase similarity analysis in Eq. (2) follows that of the previous to measure the similarity between a pair of legs. However, this analysis only compares the swing phase of each leg. The total swing state similarity in the formula only counts if a pair of legs is in the swing phase at the same time. These analyses are applied to all possible pairs of legs. The light dung ball condition was also analysed using the same procedures.

## Results and Discussion

### Leg coordination of ball rolling behaviour

**Figure 2:**
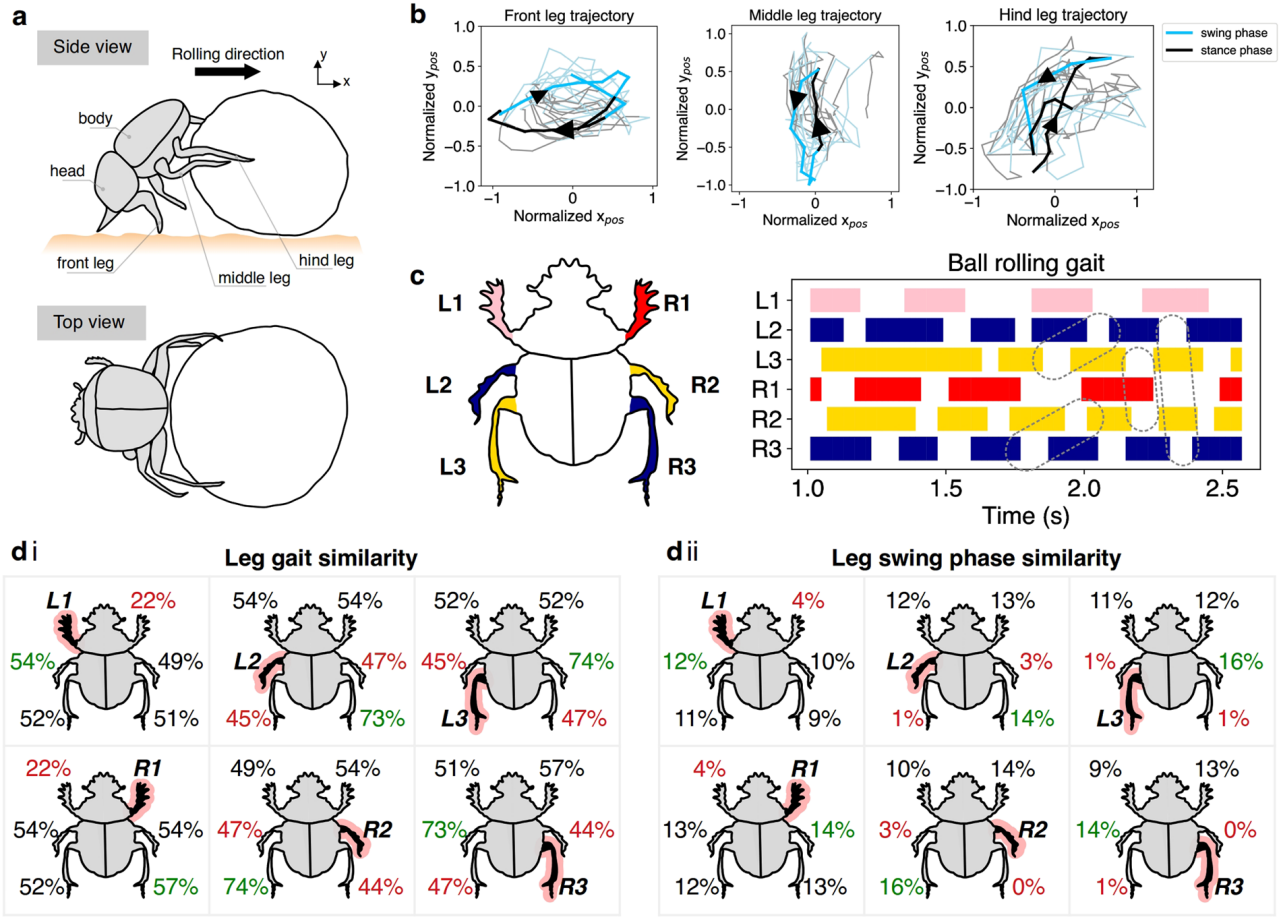
Gait patterns and leg movements of dung beetle ball rolling behaviour with leg similarity percentages. (**a**) A schematic of side and top view of the ball rolling behaviour of the dung beetle Scarabaeus (Kheper) lamarcki (see also Supplementary Movie S1). (**b**) Leg trajectories of the front, middle, and hind legs. The blue trajectory indicates the swing phase and the black trajectory the stance phase. Light blue and grey trajectories show the statistics of the leg trajectories from multiple steps. The leg trajectories were obtained from one individual run of a beetle for a duration of 5 seconds (see Supplementary Movie S1). (**c**) Rolling gait of the dung beetle with highlights of the leg coordination rules. The white spaces in the gait pattern indicate the swing phase of the leg. The dashed enclosures which cover L2 and L3 and R3 and R2 indicate the protraction wave (swing phase) on the left and right sides of the dung beetle. The vertical dashed enclosures which cover L2 and R3 and L3 and R2 indicate the overlapping of the protraction wave between the left and right sides of the dung beetle. The ball rolling gait was selected from 8 individual runs (i.e., 8 gait diagrams) for a duration of 2 seconds each. (**di**) Leg gait and (**dii**) leg swing phase similarity percentages of each pair of legs. The labelled legs in (**di)** and (**dii**) are the references for the analysis pairs. Numbers in red and green indicate the lowest and highest similarity percentages, respectively. The leg similarity percentages were calculated from 8 individual runs for a duration of 2 seconds each.

We studied the ball rolling behaviour of the dung beetle *Scarabaeus (Kheper) lamarcki*. Figure 2a shows side and top views of a ball rolling beetle. The side view (see Fig. 1c and Supplementary Movie S1) was used to extract the leg trajectories (Fig. 2b). Here we used a dung ball of 3 cm in diameter. This size has a diameter-to-dung beetle body length ratio of approximately 1:1 and a diameter-to-dung beetle’s hind leg length ratio of approximately 2:1 (for further details see the Methods section).

In typical rolling behaviour, the dung beetle’s posture is as shown in Fig. 2a. (see Supplementary Movie S1, for example). When rolling a dung ball, *S. lamarcki* use their front legs to push off the ground to generate the force required to roll a dung ball backwards. At the same time, their middle and hind legs step on the dung ball. Each leg seems to move in a steady, cyclic pattern (Fig. 2b). This is in contrast to the observation by Matthews^21^, who found that the middle leg stepped both on the ground and the dung ball. We extracted the leg trajectories by tracking the positions of the front, middle, and hind leg tips (see yellow, turquoise-blue, and green dots in Fig. 1c and Supplementary Movie S1) relative to the body (see red dot in Fig. 1c and Supplementary Movie S1) as observed from the side (Fig. 2a). For each leg trajectory, the normalized X_pos_ and Y_pos_ values in Fig. 2b were calculated by using the mean normalization method. We first subtracted the original X_pos_ and Y_pos_ values by the mean values of the X_pos_ and Y_pos_ values, respectively. Afterward, we divided the subtracted X_pos_ and Y_pos_ values by 2 times of the magnitude of range of the X_pos_ and Y_pos_ values, respectively. The front legs moved in a clockwise direction which is similar to the leg movement of an insect walking backwards on the ground^23,24^. In the stance phase, the front legs were protracted and pushed off the ground. In the swing phase, they were retracted in the air and then pushed off the ground in the new cycle of movement. The middle and hind legs seemed to have a similar movement pattern, but in an opposite direction to the front legs, i.e., they moved in a counterclockwise direction which is analogous to walking forwards on the dung ball. The legs grabbed and followed the ball in the stance phase and were retracted inward to the front in the swing phase. While the middle and hind legs showed the same movement direction, their trajectories were different (Fig. 2b). This could be due to the body tilting and/or the dynamics of the ball rolling motion disturbing the legs when touching the ball. These complex leg trajectories describe how an individual leg moves when the dung beetle performs ball rolling behaviour and are a result of intra-leg or joint coordination, which is not the main focus of this study. Instead, here, we concentrate on the inter-leg coordination that forms the basis of a “ball rolling gait”.

To do so, we extracted leg movement data with a length of two seconds from eight video recordings and performed statistical analysis to find the underlying gaits and rules. Noted that we also collect the rolling speed respected to each gait pattern (see also Supplementary Fig. S1). Figure 2c shows an extracted representative ball rolling gait and rules, highlighted in different colours. The rules highlighted in Fig. 2c are derived from leg gait similarity percentages (Fig. 2di) and leg swing phase similarity percentages (Fig. 2dii). The percentages in Figs. 2di and 1dii are the result of leg gait and leg swing phase similarity analysis (see Supplementary Methods). The leg gait similarity analysis basically compares the swing and stance phases of a pair of legs. If the gaits for a pair of legs are very similar (i.e., symmetric), they are more likely to perform the same stance and swing phases. However, if the gaits for a pair of legs are dissimilar (i.e., asymmetric), they become more likely alternate stance and swing phases. Leg swing phase similarity analysis only compared the swing phase of both legs. From the leg gait similarity analysis (Fig. 2di), our findings are summarised into two rules (highlighted in Fig. 2c):

**Rule 1**. Front legs alternately step on the ground.

**Rule 2**. Each middle leg steps similarly to its contralateral hind leg.

The first rule can be used to describe the relationship between the two front legs (L1, R1) in the gait. These legs have a low similarity percentage (22%, Fig. 2di) compared to the other legs. This shows that the two legs are more like an asymmetric gait. The rolling gait is highlighted in red and light red to show the alternation of the legs L1 and R1 (Fig. 2c). The second rule describes the synchronisation of the contralateral middle and hind legs. From the leg gait similarity percentages (Fig. 2di), we can see that the gait of L2 is highly similar to R3 (>70%) as is the gait of L3 to R2 (>70%) compared to the other legs. The blue highlight shows synchronisation between legs L2 and R3 while yellow shows the synchronisation between legs L3 and R2 (Fig. 2c). From the leg gait similarity analysis, we can identify that when a pair of legs has high leg gait similarity, the pair is very likely to swing and retain their stance at the same time. From the leg swing phase similarity analysis, we found one more rule to describe the rolling gait.

**Rule 3a**. An ipsilateral pair of middle and hind legs rarely lift together.

**Rule 3b**. Also, a contralateral pair of middle or hind legs rarely lift together.

If we consider only the group of middle and hind legs and select one reference leg, the leg on the ipsilateral and contralateral sides of the reference leg shows a low swing phase similarity percentage ($\le$3%, Fig. 2dii). In Fig. 2c, the ipsilateral (L2, L3 and R2, R3) and contralateral (L2, R2 and L3, R3) leg pairs hardly lift or swing together as we rarely observe the swing phase (white space) of the leg pairs occurring at the same time. In conclusion, a pair of legs following the third rule hardly lifts together.

Our ball rolling rules can be used to describe the inter-leg coordination of ball rolling behaviour. There are well-known leg coordination rules for describing the inter-leg coordination of locomotion behaviour proposed by Wilson^3^. Can a Wilson rule also be used to describe the leg coordination of ball rolling behaviour in the middle and hind legs interacting with the ball? One of the Wilson rules states that a wave of protraction runs from posterior to anterior. Posterior and anterior are defined with respect to the body. By observing the first two dashed enclosures in the rolling gait diagram (Fig. 2c), we can see a wave of protraction from back to front on both left and right sides of the body. On the right side, legs swing in the repeating order of R3 → R2 → R3 (if R3 is considered first) or R2 → R3 → R2 (if R2 is considered first). On the left side, legs swing in the repeating order of L3 → L2 → L3 (if L3 is considered first) or L2 → L3 → L2 (if L2 is considered first). If the protraction wave of the left and right sides of the body is fast enough, this can produce overlapping in the swing phase of legs L2 and R3 and L3 and R2 (see the two vertical dashed enclosures in Fig. 2c). The overlapping of the swing phase may be because of a coupling between the legs. This shows that locomotion and object manipulation (pushing the ball) share a common leg coordination rule:

**Rule 4**. There is a wave of protraction running to and fro within the group of middle and hind legs.

One issue that remains unclear is no gait relationship can be seen between the group of middle and hind legs and the group of front legs. When we look at the leg gait similarity percentages of legs L1 and R1 in Fig. 2di, they do not have any specific similarity in the middle and hind legs. Moreover, we cannot find any wave of protraction that connects legs L1 and R1 with their posterior legs. One possible explanation is that the front legs decouple with the group of middle and hind legs. The decoupling of the front legs may be due to differing conditions between the front, middle, and hind legs. Firstly, the different substrates (ground and ball), which the legs step on or interact with, may have different effects. Secondly, the ground reaction force acting on the front legs is higher than the middle and hind legs due to the rolling posture. This could make the front legs move slower than the other legs to maintain stability. Previous studies have also shown decoupling of insect legs in walking and searching^12–14^. It has also been observed in stick insect walking where one separate leg can adapt to walk on a treadwheel at different speeds (ranging from 1 cm/s to 12 cm/s)^12^. Stick insects also show that an individual leg performs ground searching with an asymmetric gait when crossing a gap at an average walking speed of 3 cm/s^13,14^.

### Rolling behaviour with a lighter dung ball

In the previous section, our ball rolling rules were derived from only one condition, now we would like to investigate whether the rules can also be applied to other rolling conditions, such as rolling a ball with a lighter weight. We conducted experiments to observe leg coordination in the lighter dung ball condition using a different beetle. The weights of the normal (baseline) dung ball (investigated in the earlier section) and the lighter one are 33 g and 15 g (approximately 50% of the baseline ball), respectively, while the diameter was kept constant at 3 cm.

**Figure 3:**
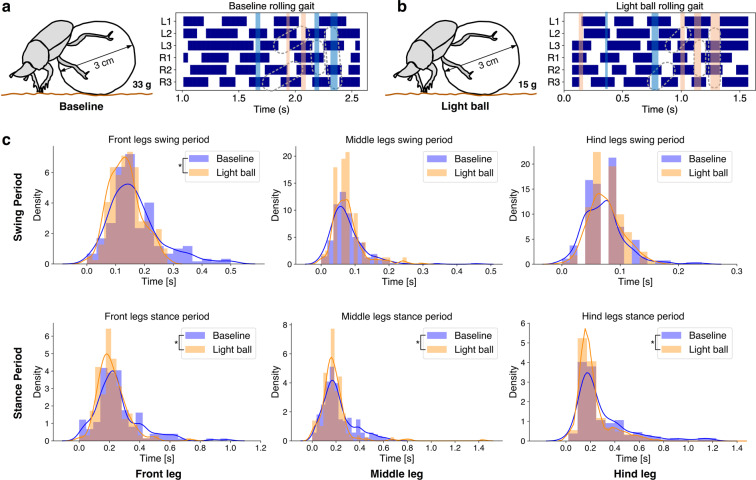
Comparison between the baseline and lighter dung ball conditions. (**a**) Rolling gait of the baseline condition. (**b**) Rolling gait of the lighter dung ball condition. Further details of the leg gait similarity percentages can be seen in Supplementary Fig. S2. The rolling gaits (**a**,**b**) are a mixture of at least a typical tripod gait (highlighted by blue bars) and the gait involving the ipsilateral front and middle legs and the contralateral hind leg (highlighted by orange bars). (**c**) Normalised density curve (using kernel density estimation) and histogram of the stance and swing phases of the baseline and lighter dung ball conditions (*p < 0.05 two-tailed, Mann-Whitney U test). (see also Supplementary Fig. S3 and Table S1). In each condition, we gathered a total of 21 individual runs from five different beetles with a duration of 2 seconds each.

The rolling gaits of the baseline and lighter ball conditions are shown in Figs. 3a,b. The representative rolling gait of the lighter ball was selected from five gait diagrams (i.e., five runs) with a length of 2 seconds each. We used the proposed ball rolling rules obtained from the baseline ball to describe the rolling gait of the lighter ball. In the rolling gait of the lighter ball (Fig. 3b), the protraction wave pattern of the hind and middle legs, based on our fourth rule, is observed. We observe the protraction waves of R3 $\,\to \,$R2 $\,\to \,$R3… and also L3 $\,\to \,$L2 $\,\to \,$L3… which are highlighted with the first two dashed enclosures (Fig. 3b). We also observe the overlapping in the swing phase of legs L2 and R3 and L3 and R2 (see the two vertical dashed enclosures in Fig. 3b). We performed leg gait and leg swing phase similarity analysis on the light ball for comparison with the baseline condition. The complete result can be seen in the supplemental information (see Supplementary Fig. S2). From the leg gait similarity analysis, we can observe the alternation of the front legs (rule 1). The gait of leg L2 is similar to leg R3 while leg L3 is similar to leg R2 (rule 2). The swing phase of legs L2 and L3 did not overlap as well as for legs R2 and R3 (rule 3a). The swing phase of legs L2 and R2 did not overlap as well as for legs L3 and R3 (rule 3b). Therefore, the proposed ball rolling rules (1–3) can be used to explain the inter-leg coordination of the ball rolling behavior in both conditions.

According to the leg gait similarity of the baseline condition (Fig. 2di), both front legs do not have a greater similarity with the same ipsilateral legs; i.e., L1 has a greater similarity with its ipsilateral middle leg L2 (54%) while R1 has a greater similarity with its ipsilateral hind leg R3 (57%). However, in the light ball condition (Supplementary Fig. S2a), both front legs have a greater similarity with the same ipsilateral legs. The similarity of the front leg and its ipsilateral middle leg is higher than its diagonal middle leg. The gait of leg L1 is more similar to leg L2 (63%) than leg R2 (43%) (Supplementary Fig. S2a), while in baseline condition leg L1 is slightly similar to leg L2 (54%) than leg R2 (49%) (Fig. 2di). In addition, the gait of leg R1 is also more similar to leg R2 (63%) than leg L2 (43%) (Supplementary Fig. S2a), while in baseline condition leg R1 is similar to leg R2 (54%) as same as leg L2 (54%) (Fig. 2di). This implies that the inter-leg coordination patterns of the baseline and light ball conditions are different. The different coordination patterns may cause by different coupling strengths between the front legs and their ipsilateral middle and hind legs. The coupling strength between the front legs and their ipsilateral middle legs in the light ball condition might be strong (i.e., they are tightly coupled) while in the baseline condition the coupling strength between the front and ipsilateral middle legs is weaker (i.e., they are loosely coupled or can be decoupled). Although the leg coordination patterns of both conditions are different, we can still observe common gaits embedded in the ball rolling gaits. Figure 3a,b show a mixture of at least a typical tripod gait (pairing of L1(R1), R2(L2), and L3(R3), highlighted by blue bars) and the gait involving the ipsilateral front (L1(R1)) and middle legs (L2(R2)) and the contralateral hind leg (R3(L3)) (highlighted by orange bars). We also see the protraction waves within the group of middle and hind legs (as described by rule 4). The proportion of the observed common gaits is different in both conditions. In the light ball condition, the dung beetles are more likely to perform the gait involving the ipsilateral front and middle legs and the contralateral hind leg than a tripod gait and vice versa in the baseline condition. In fact, during ball rolling, a rolling gait is more complex. It can involve many different gaits depending on many factors, like the speed and dynamics of ball rolling, terrain, size and weight of beetle, size and weight of dung ball, rolling posture, body and/or ball tilting, etc.

Since these two conditions seem to have slightly different gait patterns, we further analysed the duration of the stance and swing phases of ball rolling behaviour. In each condition, we gathered a total of 21 rolling gait patterns from five different beetles with a duration of 2 seconds each. The stance and swing phases were then collected from these gait patterns for analysis. Figure 3c shows the normalised density curve and histogram of the stance and swing phases, grouped into the front, middle, and hind legs. The sample size, max, median, mode, mean, standard deviation, and variance values of the swing and stance phases for the baseline and light ball conditions are shown in Supplementary Table S1. The normality of each density curve was investigated through quantile-quantile (q-q) plots (Supplementary Fig. S3). The q-q plot from each condition shows a similar pattern. For most of the q-q plots, the data are highly concentrated in the left tail and highly spread at the right tail of the plots. The data pattern implies that the distributions are skewed right and the data highly spread at the right tail. (Supplementary Fig. S3). Consequently, the distributions are more appropriate to consider as a non-standard normal distribution. Since the distributions of different beetles between baseline and light ball conditions are not normal and also uncorrelated, it is appropriate to use the Mann-Whitney U test for statistics of each condition. The results show that there are factors that significantly affect the stance and swing phases of the front legs as well as the stance phase of the middle and hind legs of the ball rolling behavior (p < 0.05) (see Fig. 3c and Supplementary Table S1). One of the factors could be the weight of the ball and another one could be the difference between individual beetles. Each beetle has different mass, size, strength, movement/rolling speeds which can affect the duration of stance and swing phases. From our analysis shown in Supplementary Fig. S4, we cannot find a clear correlation between the body mass and size, which is here represented or estimated by the body width, and the duration of stance and swing phases. We only observe a very weak correlation between the rolling speed and the duration of stance and swing phases where the variance of the distribution in a slow rolling speed seems to be higher than the variance in a fast rolling speed. From this point, we cannot conclude that the mass, size, and rolling speed could affect the duration of stance and swing phases. We believe that there are also other factors, like the dynamics of ball rolling, including rolling posture, body and/or ball tilting, step and stride lengths, that can affect the duration of stance and swing phases. Thus, further investigation on this is required. The mode values (value that appears most often) of each density curve from each condition are quite close together which can be seen in Fig. 3c. The difference in the mode values of both stance and swing phases for each condition ranges from 0.0 to 0.02 seconds (see Supplementary Table S1). However, when comparing the distribution variance, the baseline condition (heavier ball weight) seems to have a higher variance than the light ball. The higher variance in this baseline condition is basically more pronounced in the movement of the front legs than the middle and hind legs, except for the variance in the stance phase of the hind legs which is relatively higher compared to the others. The high variance of the front legs may be due to the ball weight which affects the momentum and the dynamics of ball rolling behaviour. The high variance of the hind legs may be affected by their long stance or long ball holding period (see Supplementary Table S1).

## Conclusions

To roll a ball backwards, the leg trajectories of the dung beetle show that the front legs move as it walks backwards on the ground while the middle and hind legs move as it walks forwards; thereby pushing the dung ball. We also analysed the rolling gait using a statistical approach to find the relationship between legs. From the gait pattern analysis, we identified four rules to describe leg coordination during ball rolling. The first rule describes the alternation of the front legs. The second rule describes the symmetric gait between the middle legs and their contralateral hind legs. The third rule describes that a pair of middle and hind legs on either the ipsilateral or contralateral side rarely lift together. The fourth rule is inspired by Wilson’s rule^3^ describing a protraction wave in cockroaches. This rule can also be used to describe the protraction wave in the middle and hind legs of the dung beetle during ball rolling. Interestingly, we found that while rolling a ball backwards, the front legs are decoupled or loosely coupled from the other legs, resulting in non-standard gaits. We also performed the experiments with a lighter dung ball where the proposed leg coordination rules can also be applied to describe the lighter-ball rolling gait pattern.

Taken together, our results provide insight into the principles of leg coordination in the beetle’s ball rolling behaviour and its underlying rules. The proposed rules can be used as a basis for further investigation into ball rolling behaviours on more complex terrain (e.g., uneven terrain and slopes) as well as to control bio-inspired ball rolling robots.

## Supplementary information

Supplementary information.

## References

[1] Cruse, H., Dürr, V., Schilling, M. & Schmitz, J. Principles of Insect Locomotion. In *Spatial Temporal Patterns for Action-Oriented Perception in Roving Robots* (eds. Arena, P. & Patanè, L.) Vol. 1, 43–96, 10.1007/978-3-540-88464-4_2 (Springer Berlin Heidelberg, 2008).

[2] Hughes GM (1952). The Co-Ordination of Insect Movements. J. Exp. Biol..

[3] Wilson DM (1966). Insect Walking. Annu. Rev. Entomol..

[4] Delcomyn F (1971). The Locomotion of the Cockroach Periplaneta Americana. J. Exp. Biol..

[5] Spirito CP, Mushrush DL (1979). Interlimb Coordination During Slow Walking in the Cockroach: I. Effects of Substrate Alterations. J. Exp. Biol..

[6] Ayali A (2015). The comparative investigation of the stick insect and cockroach models in the study of insect locomotion. Curr. Opin. Insect Sci..

[7] Graham D (1972). A behavioural analysis of the temporal organisation of walking movements in the 1st instar and adult stick insect (Carausius morosus). J. Comp. Physiol..

[8] Dean J, Wendler G (1983). Stick Insect Locomotion on a Walking Wheel: Interleg Coordination of Leg Position. J. Exp. Biol..

[9] Cruse H, Knauth A (1989). Coupling Mechanisms Between the Contralateral Legs of a Walking Insect (Carausius Morosus). J. Exp. Biol..

[10] Cruse H (1990). What mechanisms coordinate leg movement in walking arthropods?. Trends Neurosci..

[11] Dean J (1991). A model of leg coordination in the stick insect, Carausius morosus. Biol. Cybern..

[12] Bässler U, Büschges A (1998). Pattern generation for stick insect walking movements—multisensory control of a locomotor program. Brain Res. Rev..

[13] Durr V (2001). Stereotypic leg searching movements in the stick insect: kinematic analysis, behavioural context and simulation. J. Exp. Biol..

[14] Blaesing B, Cruse H (2004). Stick insect locomotion in a complex environment: Climbing over large gaps. J. Exp. Biol..

[15] Grabowska M, Godlewska E, Schmidt J, Daun-Gruhn S (2012). Quadrupedal gaits in hexapod animals – inter-leg coordination in free-walking adult stick insects. J. Exp. Biol..

[16] Dallmann CJ, Hoinville T, Dürr V, Schmitz J (2017). A load-based mechanism for inter-leg coordination in insects. Proc. R. Soc. B Biol. Sci..

[17] Pfeffer SE, Wahl VL, Wittlinger M (2016). How to find home backwards? Locomotion and inter-leg coordination during rearward walking of Cataglyphis fortis desert ants. J. Exp. Biol..

[18] Khaldy, L., Tocco, C., Byrne, M., Baird, E. & Dacke, M. Straight-line orientation in the woodland-living beetle Sisyphus fasciculatus. *J. Comp. Physiol*. A 1–9, 10.1007/s00359-019-01331-7 (2019). 10.1007/s00359-019-01331-7 PMC7192865 30955076

[19] Smolka J, Byrne MJ, Scholtz CH, Dacke M (2013). A new galloping gait in an insect. Curr. Biol..

[20] Ignasov J (2018). Bio-inspired design and movement generation of dung beetle-like legs. Artif. Life Robot.

[21] Matthews EG (1963). Observations on the ball-rolling behavior of Canthon pilularius (L.)(Coleoptera, Scarabaeidae). *Psyche A*. J. Entomol..

[22] Schneider CA, Rasband WS, Eliceiri KW (2012). NIH Image to ImageJ: 25 years of image analysis. Nat. Methods.

[23] Graham D, Epstein S (1985). Behaviour and Motor Output for an Insect Walking on a Slippery Surface: II. Backward Walking. J. Exp. Biol..

[24] Rosenbaum P, Wosnitza A, Büschges A, Gruhn M (2010). Activity Patterns and Timing of Muscle Activity in the Forward Walking and Backward Walking Stick Insect Carausius morosus. J. Neurophysiol..

