# Author Correction: Rules for the Leg Coordination of Dung Beetle Ball Rolling Behaviour

**DOI:** 10.1038/s41598-020-74831-1

**Published:** 2020-10-20

**Authors:** Binggwong Leung, Nienke Bijma, Emily Baird, Marie Dacke, Stanislav Gorb, Poramate Manoonpong

**Affiliations:** 1 Bio-Inspired Robotics and Neural Engineering Lab, School of Information Science and Technology, Vidyasirimedhi Institute of Science and Technology, Rayong, 21210 Thailand; 2 Functional Morphology and Biomechanics, Zoological Institute, Kiel University, Kiel, 24118 Germany; 3 Division of Functional Morphology, Department of Zoology, Stockholm University, Stockholm, SE, 10691 Sweden; 4 Department of Biology, Lund Vision Group, Lund University, Sölvegatan 35, 223 62 Lund, Sweden; 5 Embodied AI and Neurorobotics Lab, SDU Biorobotics, The Mærsk Mc-Kinney Møller Institute, The University of Southern Denmark, Odense, 5230 Denmark

Correction to: *Scientific Reports* https://doi.org/10.1038/s41598-020-66248-7, published online 09 June 2020

Supplementary Movie S1, which depicts the typical rolling behaviour of the dung beetle, was omitted from this Article. This file is linked to this correction notice.

## Supplementary information

Supplementary Movie S1.

