# Supplementary material for: Rules for the Leg Coordination of Dung Beetle Ball Rolling Behaviour
Source: Sci Rep. 2020 Jun 9;10:9278. doi: 10.1038/s41598-020-66248-7 (PMC7283283; doi:10.1038/s41598-020-66248-7)
Supplement: Supplementary file 1 — Supplementary information. [file 41598_2020_66248_MOESM1_ESM.pdf]

Supplementary Information:

Rules for the Leg Coordination of Dung Beetle Ball Rolling Behaviour

Binggwong Leung, Nienke Bijma, Emily Baird, Marie Dacke, Stanislav Gorb, and Poramate Manoonpong

Supplementary Methods

Dung beetles and ball rolling experiments

Dung beetles of the species *Scarabaeus (Kheper) lamarcki* were collected from the "Stonehenge" game farm in South Africa (24.32°E, 26.39°S) during November 2017. The beetles were kept in sand-filled plastic boxes and fed cow dung ad libitum. The experiments were performed outdoors under natural skylight conditions at Thornwood Lodge (near Bela-Bela: 24.46°S, 28.00°E). To characterise the gaits, beetles were filmed at 50 frames per second using video cameras (Sony RX10III) as they walked on the natural ground in the field. Balls of different weights (but with the same diameter) were prepared by embedding plasticine into them; either styrofoam (light balls defined here as the lighter ball condition) or little stones (heavy balls defined here as the baseline ball condition). The balls were then covered by the dung used by the beetles in their natural environment.

Tools for ball rolling gait pattern analysis

In this work, we use Python programming language with standard libraries to perform ball rolling gait pattern analyses, and NumPy and Pandas libraries for data manipulation. In addition, we use Matplotlib and Seaborn for data visualisation. The normalised density curves in relation to Figure 3c are plotted using kernel density estimation from the Seaborn library. We also use default parameters for the kernel density estimation from the Seaborn library to create the density curves. For statistical tests, we use Scipy.stats library to create quantile-quantile plots and perform the Mann-Whitney U test on the data of stance and swing periods.

Supplementary Figures

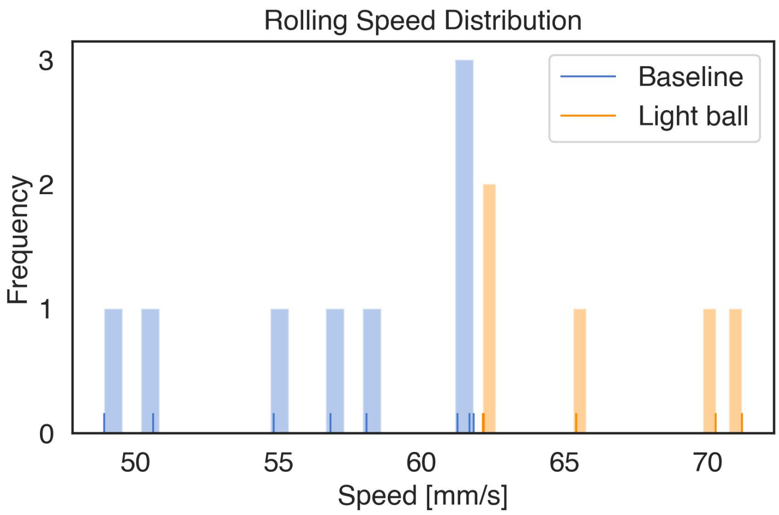

Supplementary Figure S1: Rolling speed distribution of baseline and light ball conditions used for gait pattern analysis with respect to Figure 2c, 2di, 2dii and Figure 3a, 3b.

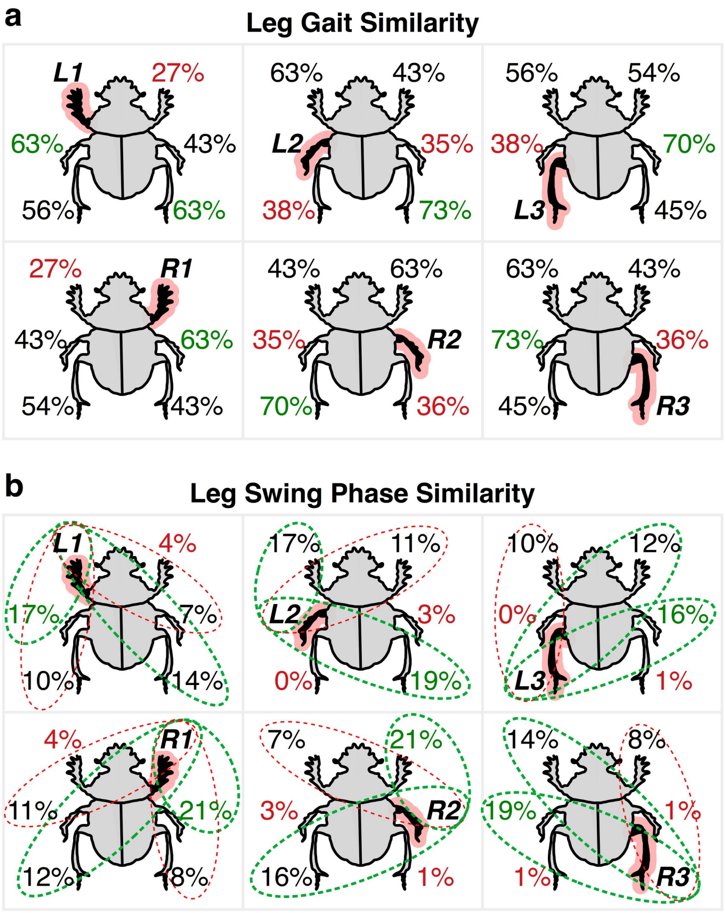

Supplementary Figure S2: Leg gait similarity percentages of the light ball condition of dung beetle ball rolling behaviour, in relation to Figure 3b. (**a**) Leg gait similarity percentages of each pair of legs. (**b**) Leg swing phase similarity percentages of each pair of legs. The labelled legs in (**a**) and (**b**) are the reference for the analysis pairs. Numbers in red and green indicate the lowest and highest similarity percentages, respectively. Green ellipse means high similarity where the legs can swing together at the same time. Red ellipse means low similarity where the legs can show an overlapping in the swing phase.

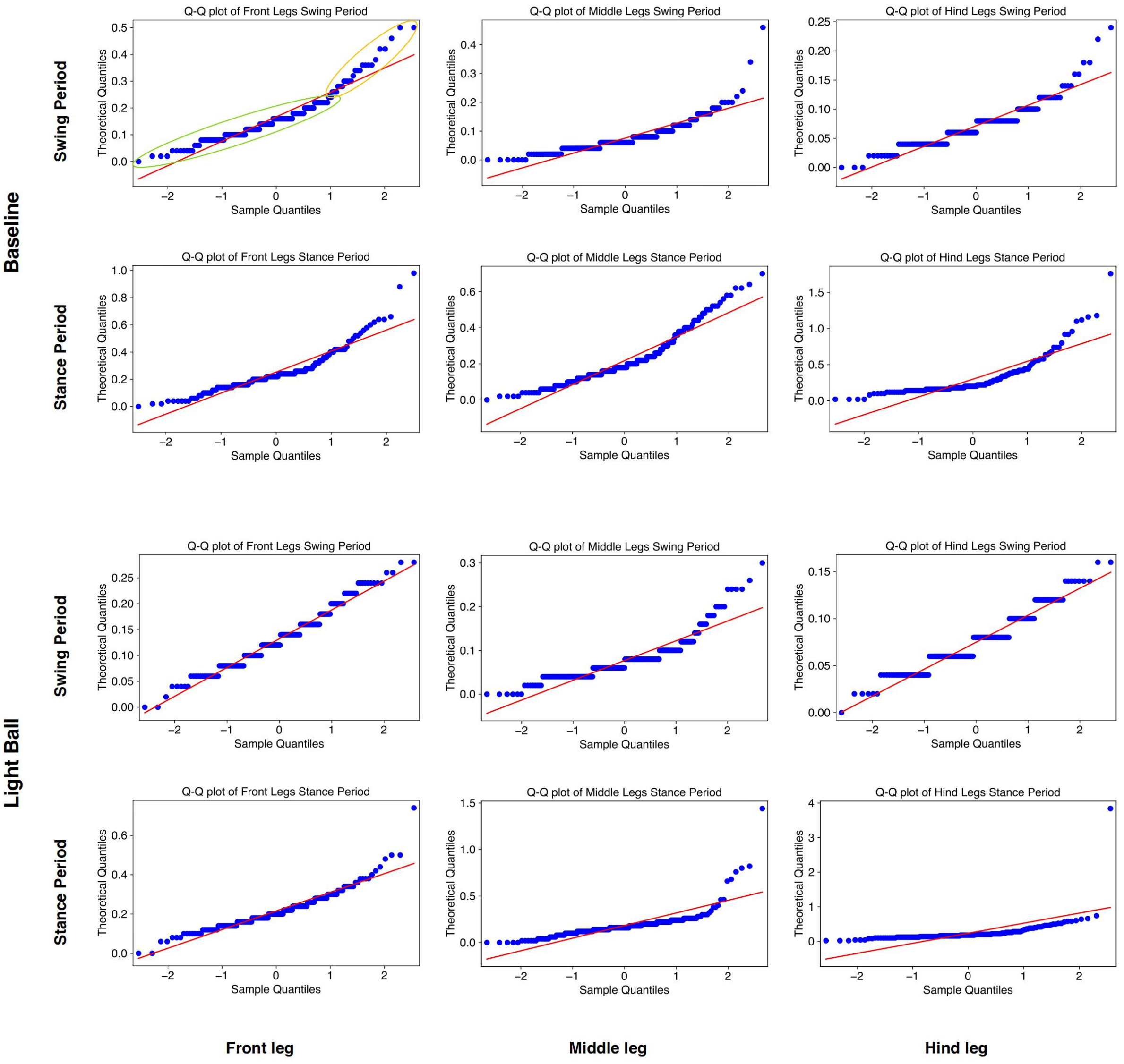

Supplementary Figure S3: Quantile-quantile plots of the stance and swing phases from baseline and light ball conditions of dung beetle ball rolling behaviour, related to Figure 3c. The quantile-quantile plots show the normality of the data distribution of the stance and swing phases from baseline (heavier ball) and light ball conditions. If the data lie perfectly on the red diagonal line in the q-q plot, the distribution can be interpreted as the standard normal distribution. In the q-q plot of the front leg swing phase of the baseline condition, we draw ellipses to highlight the data distribution. The green ellipse covers the data which are highly concentrated in the left tail of the distribution while the orange ellipse covers the data which are highly spread at the right tail of the distribution. Thus, we can interpret from the distribution that it is skewed right and the data highly spread at the right tail. The other plots also show almost the same pattern.

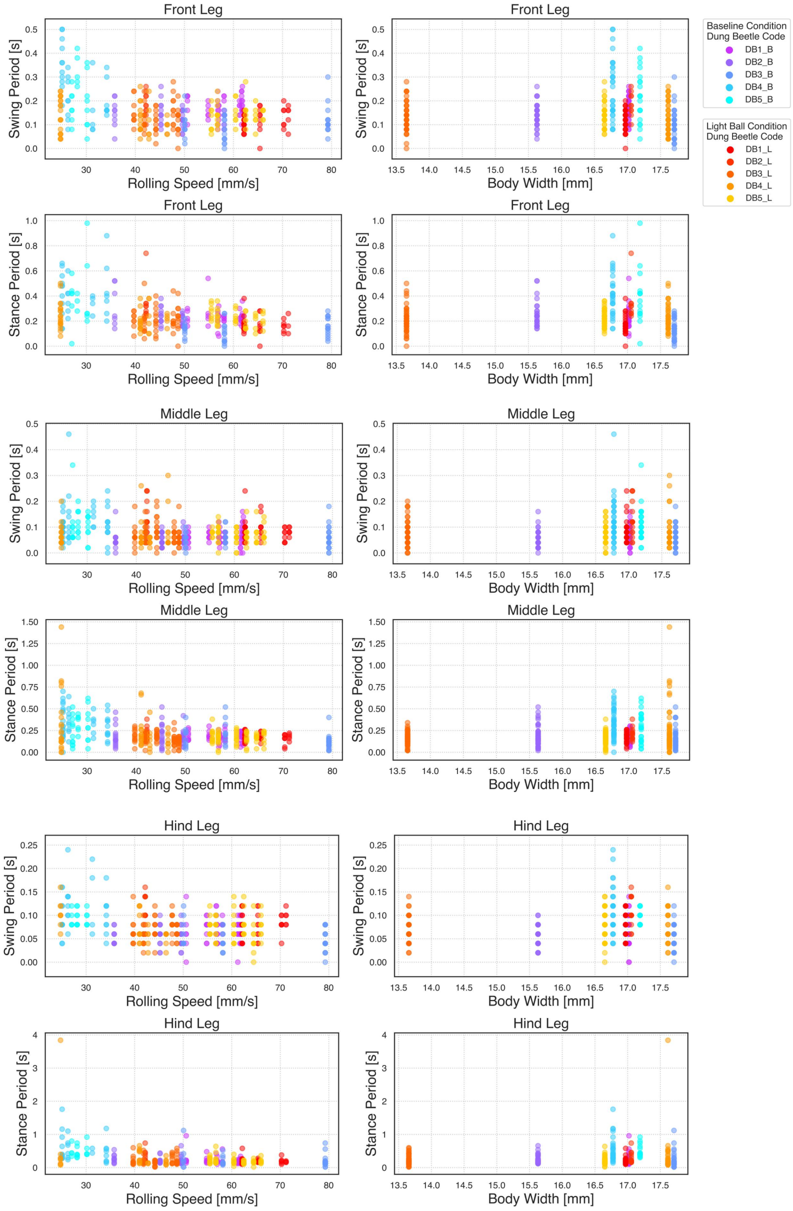

Supplementary Figure S4: The scatter plot between the rolling speed, the width of dung beetle of each run and the time duration of swing and stance phases of both baseline and light ball conditions with respect to Figure 3c. The stance and swing phases of each condition are gathered from 21 baseline/light rolling gait patterns (i.e., 21 individual runs from 5 different beetles with a duration of 2 seconds of each run). Each color represents the distribution of each individual beetle. Dung Beetle Code refers to the dung beetles that were used in the experiments in the baseline and light ball conditions. DB1_B, DB2_B, ..., DB5_B are the dung beetle number 1, 2, …, and 5 in the baseline condition and DB1_L, DB2_L, ..., DB5_L are the dung beetle number 1, 2, …, and 5 in the in the light ball condition. Width of each dung beetle is measured in the middle of the body from the left to the right side of the body. Speed is calculated from dividing the distance between the starting point and the endpoint of each run by rolling time.

Supplementary Table

Supplementary Table S1: Statistical value of the baseline and light ball conditions of dung beetle ball rolling behaviour, related to Figure 3c. The table show sample size (one sample means one stance or swing duration), max, median, mode, mean, standard deviation, and variance values of the swing and stance periods for the baseline and light ball conditions. The median, mode, mean, standard deviation, and variance values are shown by the colour scales, ranging from lowest (red), median (yellow) to highest (green). The p values of the Mann-Whitney U test of the stance and swing periods between the baseline and light ball conditions are shown.

		**Front Legs**		**Middle Legs**		**Hind Legs**	
		Swing Period(s)	Stance Period(s)	Swing Period(s)	Stance Period(s)	Swing Period(s)	Stance Period(s)
**Baseline**	N	179	159	260	239	201	174
	max	0.5	0.98	0.46	0.7	0.24	1.76
	median	0.16	0.22	0.06	0.18	0.06	0.2
	mode	0.16	0.24	0.06	0.18	0.08	0.16
	mean	0.167	0.253	0.076	0.218	0.072	0.3
	SD	0.091	0.155	0.052	0.134	0.035	0.246
	variance	0.008	0.024	0.003	0.018	0.001	0.061
**Light ball**	N	197	181	265	252	211	190
	max	0.28	0.74	0.3	1.44	0.16	3.84
	median	0.12	0.2	0.06	0.16	0.08	0.18
	mode	0.14	0.18	0.08	0.16	0.06	0.16
	mean	0.132	0.216	0.077	0.183	0.075	0.236
	SD	0.056	0.095	0.045	0.135	0.029	0.291
	variance	0.003	0.009	0.002	0.018	0.001	0.085
P value (Mann-Whitney U test, two tailed, alpha = 0.05)		0.0002	0.0273	0.1795	0.0014	0.0565	0.0005
